# Impact of cancer mutational signatures on transcription factor motifs in the human genome

**DOI:** 10.1186/s12920-019-0525-4

**Published:** 2019-05-20

**Authors:** Calvin Wing Yiu Chan, Zuguang Gu, Matthias Bieg, Roland Eils, Carl Herrmann

**Affiliations:** 1 Division of Theoretical Bioinformatics, German Cancer Research Center (DKFZ), Heidelberg, 69120 Germany; 2 Faculty of Biosciences, Heidelberg University, Heidelberg, 69120 Germany; 3 Center for Digital Health, Berlin Institute of Health (BIH), Berlin, 10178 Germany; 4 Health Data Science Unit, Medical Faculty University Heidelberg and BioQuant, Heidelberg, 69120 Germany

**Keywords:** SNV, Cancer, Mutational signature

## Abstract

**Background:**

Somatic mutations in cancer genomes occur through a variety of molecular mechanisms, which contribute to different mutational patterns. To summarize these, mutational signatures have been defined using a large number of cancer genomes, and related to distinct mutagenic processes. Each cancer genome can be compared to this reference dataset and its exposure to one or the other signature be determined. Given the very different mutational patterns of these signatures, we anticipate that they will have distinct impact on genomic elements, in particular motifs for transcription factor binding sites (TFBS).

**Methods:**

We used the 30 mutational signatures from the COSMIC database, and derived a theoretical framework to infer the impact of these signatures on the alteration of transcription factor (TF) binding motifs from the JASPAR database. Hence, we translated the trinucleotide mutation frequencies of the signatures into alteration frequencies of specific TF binding motifs, leading either to creation or disruption of these motifs.

**Results:**

Motif families show different susceptibility to alterations induced by the mutational signatures. For certain motifs, a high correlation is observed between the TFBS motif creation and disruption events related to the information content of the motif. Moreover, we observe striking patterns regarding for example the Ets-motif family, for which a high impact of UV induced signatures is observed. Our model also confirms the susceptibility of specific transcription factor motifs to deamination processes.

**Conclusion:**

Our results show that the mutational signatures have different impact on the binding motifs of transcription factors and that for certain high complexity motifs there is a strong correlation between creation and disruption, related to the information content of the motif. This study represents a background estimation of the alterations due purely to mutational signatures in the absence of additional contributions, e.g. from evolutionary processes.

**Electronic supplementary material:**

The online version of this article (10.1186/s12920-019-0525-4) contains supplementary material, which is available to authorized users.

## Background

With the availability of thousands of fully sequenced cancer genomes, genome-wide patterns of somatic mutations can be analyzed to search for potential driver mutations. Such an effort has been exemplified by the recent Pan-Cancer Analysis of Whole Genomes (PCAWG) initiative by the International Cancer Genome Consortium (ICGC) consortium. However, besides coding driver mutations that have been described earlier, non-coding mutations have been under increased scrutiny, in search for additional non-coding drivers, given the extensive number of these mutations in non-coding genomic regions. Several modes of actions can be identified for these mutations, the most likely ones being mutations affecting regulatory elements such as transcription factor binding sites (TFBS), altering in one way or the other (creation or disruption) the binding motif. A spectacular example was identified in several cancer entities involving the promoter of the *TERT* gene, in which two recurrent mutations have been shown to create new binding sites for Ets-family transcription factors, leading to a strong over-expression of the *TERT* oncogene [1, 2]. Another example was found in T-ALL, in which a mutation creating a new binding site for MYB leads to the appearance of a super-enhancer driving over-expression of the TAL1 oncogene [3]. Besides these few non-coding drivers, cancer genomes are loaded with thousands of mutations which are termed passenger, as they cannot be individually related to molecular phenotypes, as in the previous cases. However, several studies have shown that these putative passengers contribute to an overall mutational load in the cancer genome, and can, collectively, have an impact [4, 5]. Hence, it is of importance to understand the overall patterns of non-coding mutations, besides the few driving examples.

Patterns of somatic mutations have been analyzed by defining so-called *mutational signatures*, based on a dimensional reduction approach focusing on the patterns of trinucleotide alterations. The 96 possible types of trinucleotide mutations were summarized into a reduced number of signatures, which each describe a different mutational bias [6]. Some of these mutational signatures can be related to specific mutational processes such as APOBEC mutations, nucleotide mismatch repair or various carcinogens. Recently, nucleotide excision repair (NER), which is related to one mutational signature, has been related to specific patterns of mutations within TFBS in cancer genomes [7]. Once these overall signatures are available, the exposure of cancer types or of individual cancer genomes can be determined. Hence, for example, there is a clear association between the signature related to ultra-violet light and melanoma [8].

In this study, we assess the impact of mutational signatures on motifs of transcription factor binding sites. In particular, we search to understand how a particular mutational signature impacts the large collection of transcription factor binding site motifs. Our objective is to establish, for each signature, a catalogue of binding motif creation and disruption frequencies, which would correspond to an expected background effect of the mutational patterns, in the absence of any additional effect such as selective processes. Our goal is thus to translate the mutational signatures based on trinucleotide alterations into signatures of motif alteration. The result provides a theoretical framework as a baseline model for transcription binding site alteration analysis in cancer genomes.

## Method

The following regulatory impact analysis is based on the 30 trinucleotide mutational signatures described by Alexandrov et al. [6]. These mutational signatures were downloaded from the COSMIC database (https://cancer.sanger.ac.uk/cosmic/signatures). Each of the 30 mutational signature contains the normalized mutational probability across the 96 types of point mutation in a trinucleotide context. In the remaining of this paper the mutational signature is represented by the 30 x 96 mutational signature matrix *S*_*M*_ and mutational signature vector $\vec {s}_{v}(s_{i})$ as follows:

1 $$ {}S_{M} \,=\, \left[\!\!\begin{array}{c} \leftarrow Trinucleotide\;signature\;1 \rightarrow\\ \leftarrow Trinucleotide\;signature\;2 \rightarrow\\ \vdots\\ \leftarrow Trinucleotide\;signature\;30 \rightarrow\\ \end{array}\!\!\right]\!\! =\!\! \left[\begin{array}{c} \vec{s}_{v}(s_{1})\\ \vec{s}_{v}(s_{2})\\ \vdots\\ \vec{s}_{v}(s_{30})\\ \end{array}\right]  $$

2 $$ {}S_{M} = \left[\begin{array}{cccc} Pr(m_{1}|s_{1}) & Pr(m_{2}|s_{1}) & \dots & Pr(m_{96}|s_{1})\\ Pr(m_{1}|s_{2}) & Pr(m_{2}|s_{2}) & \dots & Pr(m_{96}|s_{2})\\ \vdots & \vdots & \ddots & \vdots \\ Pr(m_{1}|s_{30}) & Pr(m_{2}|s_{30}) & \dots & Pr(m_{96}|s_{30})\\ \end{array}\right]  $$

where *m*_*i*_ corresponds to the 96 possible trinucleotide mutations (eg. A[C>A]A, A[C>G]A,... etc.). This mutational signature matrix is denoted as the *trinucleotide mutational signature* in the remaining part of this article.

### Transcription Factor Motif Alteration Signature

**Fig. 1:**
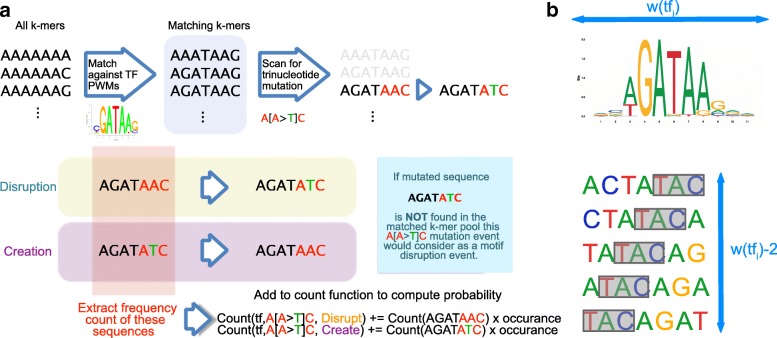
Worflow of the method: **a** Frequency counting procedure; **b** Transcription factor binding motif alteration signature analysis workflow

The procedure of computing the motif binding alteration probability is illustrated in Fig. 1.

We define *Pr*(*a*|*tf*_*i*_,*m*_*j*_) as the probability for a motif *tf*_*i*_ to undergo an alteration *a* (i.e. creation or disruption) given a trinucleotide mutation *m*_*j*_. A disruption event is defined as a mutation that turns a binding site into a non-binding site, and a creation event corresponds to the reverse effect. The probability of a transcription factor motif alteration event is computed by assessing the p-value of a given sequence being a binding site before and after the mutation. The 512 JASPAR 2016 vertebrate position weight matrices (PWM) of length 6 to 19 are used to compare the impact in binding affinity due to a point mutation [9]. The p-value of a sequence is evaluated using matrix-scan of the Regulatory Sequence Analysis Toolbox (RSAT) to compute the p-value [10]. In this study, a p-value of p=0.001 is set as the binding threshold. For a given transcription factor binding PWM of width *k*, all possible k-mer sequences are scanned to compute the mutational statistics. For a PWM of *k*=19, it requires scanning a total of 4^19^=274,877,906,944 sequences for all the 19mers. The search space of binding sequences can be reduced in half by combining all the reverse complementary sequences.

For each PWM *tf*_*i*_ of width *k*, we enumerate all possible k-mers (considering a k-mer and its reverse complement as the same motif) and, using matrix-scan with the parameters described above, we separate the set of k-mers into binding and non-binding k-mers. Then, the alteration probability is computed by mutating each of the trinucleotide in the matching k-mer according to the mutation type *m*_*i*_ and search for the corresponding mutated sequence. A disruption event is identified if the mutated sequence is not found in the list of binding k-mers. Conversely, a creation event is identified if a non-binding k-mer is turned into a binding k-mer. The current matched binding sequence is considered as the reference sequence for a motif disruption event and alternative sequence in the motif creation event and vice versa.

In order to count these events in the human genome, all possible k-mers are extracted from the human genome (version hg19) and their occurrences are counted for *k*=6 to *k*=19. The count of each reference sequence in the human genome is recorded according to the type of trinucleotide mutation and the type of alteration event (see pseudo-code in Additional File 4: Figure S4). The probability *Pr*(*a*|*tf*_*i*_,*m*_*j*_) can be obtained by normalizing the counts by the total number of reference trinucleotide *c*_*hg*19_(*m*_*j*,*ref*_) for the reference trinucleotide *m*_*j*,*ref*_ of a given mutation type *m*_*j*_ of a *tf*_*i*_ PWM width of length *w*(*tf*_*i*_). The count normalization factor for alteration probability computation is illustrated in Fig. 1b. For each trinucleotide in the genome, it is compared *w*(*tf*_*i*_)−2 times. Therefore, the total count detected should be divided by the number of trinucleotides in the genome multiplied by *w*(*tf*_*i*_)−2 to obtain the alteration probability.

3 $$ Pr(a|tf_{i},m_{j}) = \frac{c(a|tf_{i},m_{j})}{c_{hg19}(m_{j,ref}) \cdot (w(tf_{i}) - 2)}  $$

Importantly, we also need to determine the binding affinity of k-mers which do not occur in the human genome for the motif creation event, as a mutation could turn a k-mer into one which does not occur in the reference genome. However, for the disruption probability computation, the k-mers search space for genomic k-mers count can be drastically reduced by considering only k-mers occurring in the human genome, which dramatically reduces the search space for *k*>13. The motif alteration probability of a given transcription factor PWM and trinucleotide is stored in the motif alteration probability matrix,

4 $$ {}\Phi_{A|TF,M} = \left[\begin{array}{cccc} Pr(a|tf_{1},m_{1}) & Pr(a|tf_{1},m_{2}) & \dots & Pr(a|tf_{1},m_{96})\\ Pr(a|tf_{2},m_{1}) & Pr(a|tf_{2},m_{2}) & \dots & Pr(a|tf_{2},m_{96})\\ \vdots & \vdots & \ddots & \vdots \\ Pr(a|tf_{I},m_{1}) & Pr(a|tf_{I},m_{2}) & \dots & Pr(a|tf_{I},m_{96})\\ \end{array}\right]  $$

From this, we compute the alteration probability for a mutational signature *s*_*i*_ using Bayesian inference:

5 $$\begin{array}{@{}rcl@{}}  Pr(a|tf_{j},s_{i})=\sum_{k=1}^{96} Pr(a|tf_{j},m_{k}) \cdot Pr(m_{k}|s_{i}) \end{array} $$

or, in matrix notation:

6 $$\begin{array}{@{}rcl@{}}  S_{TF}=S_{M}\;\Phi_{A|TF,M}^{T} \end{array} $$

where,

7 $$\begin{array}{@{}rcl@{}} {}S_{TF}\! =\! \left[\begin{array}{cccc} Pr(a|s_{1},tf_{1}) & Pr(a|s_{1},tf_{2}) & \dots & Pr(a|s_{1},tf_{I})\\ Pr(a|s_{2},tf_{1}) & Pr(a|s_{2},tf_{2}) & \dots & Pr(a|s_{2},tf_{I})\\ \vdots & \vdots & \ddots & \vdots \\ Pr(a|s_{30},tf_{1}) & Pr(a|s_{30},tf_{2}) & \dots & Pr(a|s_{30},tf_{I})\\ \end{array}\right] \end{array} $$

The full algorithm for computing the alteration probability is given in the Additional file 4: Figure S4.

### Analysis on Transcription Factor Motif Alteration Probability

The motif alteration probability matrix *Φ*_*TF*|*M*_ encapsulates changes in binding affinity of a given PWM under the perturbation of a single nucleotide point mutation. In order to investigate similarities in the alteration probabilities of different motifs, a hierarchical clustering of motif creation and motif disruption is performed. A Partitioning Around Medoids (PAM) clustering approach is applied to partition the 512 transcription factors using the silhouette coefficient to determine the optimal number of groups. The clustering is then compared to the TF family annotation downloaded from the JASPAR database. To further investigate the alteration probability of relevant TFs in cancer, all transcription factors present in the COSMIC cancer gene census are extracted and evaluated. The global alteration offset of these COSMIC cancer TFs are computed by subtracting the disruption probability from the creation probability.

To evaluate the similarity of motif alteration probability of multiple transcription factors, a self-organizing map (SOM) analysis is performed using the alteration probability matrix *Φ*_*TF*|*M*_ to validate the result and to gain insight into the alteration similarity among the TF motifs. For this, a 22 x 22 grid was used and resulted in a stack of 96 variable maps corresponding to the trinucleotide mutation type *m*_*k*_. The map dimension of the SOM is selected to maximally retain the resolution of the transcription factor space based on the following criterion:

8 $$ \mathop{argmax}_{w_{som}} \{w_{som}^{2}\} < N_{TF}  $$

where *w*_*som*_ is the width of the SOM, and *N*_*TF*_ is the total number of TFs. There is a total of 512 TFs in the JASPAR vertebrate 2016 database, therefore the optimal SOM dimension is *w*_*som*_=22. This allows the TFs to be distributed evenly on the map in the worst case scenario when they are equally dissimilar with respect to each other.

### Comparison with the PCAWG Dataset

We compared our motif alteration prediction based on the mutational signatures with real datasets of SNVs in cancer. For this, we used a previously published dataset from the PCAWG study, containing 2708 whole-genome sequencing (WGS) samples. We restrained ourselves to the WGS and discarded whole-exome sequencing (WES) samples as we wanted to make genome-wide predictions of alterations of TFBS motif, which lie outside of coding-regions [11]. We have listed the abbreviations of the tumor subtypes in Additional file 5: Figure S5.

In order to predict motif alterations in the PCAWG dataset, we developed a motif alteration pipeline. The pipeline is based on the matrix-scan-quick tool from the RSAT toolbox [10]. The neighborhood reference sequence of each SNV is extracted to build a second order background Markov model for PWM matching. Both the reference and alternative sequence are matched against the given PWM. For alteration detection, it is required to fulfill both of the following conditions: (1) the mutation results in a change from binding site to non-binding site (or vice-versa for creation) where a threshold of *p*≤1*e*−4 is required to consider a sequence as a TF binding site; (2) a 10-fold p-value change before and after the mutation.

The PCAWG SNV calls are first matched against the same set of JASPAR PWMs to identify possible transcription factor binding alteration sites. To produce comparable results across all cancer entities, the detection probability of each of the transcription factor alteration is computed by normalizing the detection counts with respect to the total number of SNV detected in the corresponding cancer entity.

To match our predicted frequencies of alteration based on the mutational signature to the cancer dataset, we need, for each of the cancer entities, to combine the influence of those mutational signatures that contribute to the particular cancer entity. Here, we used, for each cancer sample, the exposure matrices described in [11], based on 2708 PCAWG samples. Since the PCAWG dataset is based on a new set of 48 PCAWG mutational signatures, we mapped each of these 48 signatures to the most similar out of the 30 COSMIC signatures. Similarity was assessed using Pearson correlation across the 96 trinucleotide mutations. When several PCAWG signatures mapped to the same COSMIC signature, we summed up the exposure values corresponding to these signatures. To infer the transcription factor binding site alteration probability for one cancer entity, the transcription factor motif alteration matrix *S*_*TF*_ is multiplied with the normalized exposure matrix *E*_*PID*_ to produce the per patient exposure prediction matrix *Ψ*_*PID*_,

9 $$ \Psi_{PID} = S_{TF}^{T} \; E_{PID}  $$

10 $$\begin{array}{@{}rcl@{}} {}E_{PID} = \left[\begin{array}{cccc} e(s_{1}|pid_{1}) & e(s_{1}|pid_{2}) & \dots & e(s_{1}|pid_{482})\\ e(s_{2}|pid_{1}) & e(s_{2}|pid_{2}) & \dots & e(s_{2}|pid_{482})\\ \vdots & \vdots & \ddots & \vdots \\ e(s_{30}|pid_{1}) & e(s_{30}|pid_{2}) & \dots & e(s_{30}|pid_{482})\\ \end{array}\right] \end{array} $$

where *e*(*s*_*l*_|*pid*_*m*_) is the normalized exposure (or exposure probability) of signature *l* and patient *m*. Combining the above we have,

11 $$ {}\Psi_{PID} \,=\,\! \left[\!\begin{array}{cccc} Pr(a|pid_{1},tf_{1}) & Pr(a|pid_{2},tf_{1}) & \dots & Pr(a|pid_{482},tf_{1})\\ Pr(a|pid_{1},tf_{2}) & Pr(a|pid_{2},tf_{2}) & \dots & Pr(a|pid_{482},tf_{2})\\ \vdots & \vdots & \ddots & \vdots \\ Pr(a|pid_{1},tf_{I}) & Pr(a|pid_{2},tf_{I}) & \dots & Pr(a|pid_{482},tf_{I})\\ \end{array}\!\right]  $$

After obtaining the per patient exposure prediction matrix *Ψ*_*PID*_, the median TF alteration probability within each entity is compared against the alteration probability from the alteration detection pipeline.

### Clustering of tumor samples

Given the heterogeneity of the samples within a tumor cohort, we clustered the PCAWG samples based on their exposure values. We first used the UMAP dimensionality reduction method [12] on the table of exposure values of the 2708 samples, and then defined clusters using the hdbscan method [13], as implemented in the largeVis R-package. This defined 21 clusters containing 2360 samples, while 348 samples could not be assigned to one of these clusters. Results are shown in Additional file 3: Figure S3.

## Results

### Impact of mutational signatures on transcription factor binding motifs

**Fig. 2:**
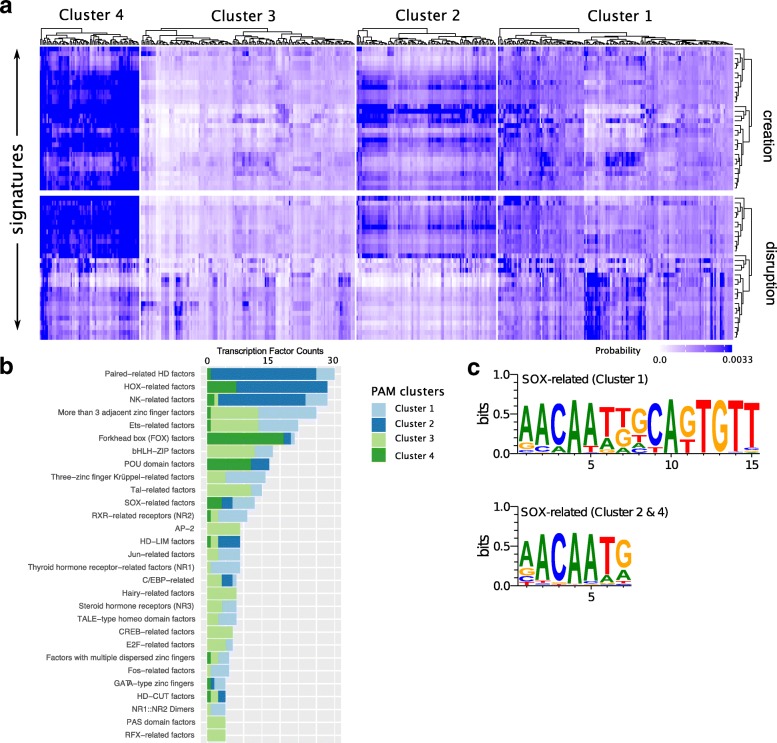
Signature driven motif alterations: **a** Motif alteration signatures heatmap where the vertical axis corresponds to the 30 mutational signatures and the horizontal axis corresponds to the 512 motifs in JASPAR database. A *k*_*clust*_=4 PAM clustering performed over the TF alteration probability; **b** Cluster count statistics of each TF families with at least 5 members; **c** Familial binding profile of SOX-related motifs associated with PAM clusters

We computed the motif alteration signature using the conditional probability between the transcription factor alteration probability and the trinucleotide mutational signature as described in Eq. 5 and Eq. 6. The predicted alteration probabilities for all TF motifs and all 30 COSMIC signatures are given in Additional file 6: Figure S6. The motif alteration results across all 512 JASPAR motifs are shown in Fig. 2a for motif creation (top) and motif disruption (bottom, see Additional file 1: Figure S1 for a more detailed representation including signature names and information on TF families.). To capture similar patterns between transcription factor motifs, we applied a PAM clustering to the set of 512 motifs. The silhouette coefficient shows a local maximum at *k*_*clust*_=4 indicating that the optimal number of clusters is 4.

The four clusters show a completely different behavior under the mutation signatures; whereas cluster 3 shows a low sensitivity to any of the mutational signatures (with some exceptions), cluster 4 on the other hand seems to be strongly impacted both by motif creation and disruption. Interestingly, comparing the disruption and creation heatmaps, we observe that the creation sensitivity for cluster 4 is high over all signatures, whereas we observe two groups of signatures for the disruption, with different impacts. We then studied the composition of TF motifs of each cluster. As expected, none of the clusters is dominated by a single TF family (Fig. 2b). However, Fig. 2b shows that some motif families are preferentially associated to one of the clusters. Forkhead motifs are in their vast majority associated with cluster 4 and show a high sensitivity to all mutational signatures. We also observe obvious mutual exclusivity between the cluster 1 and 2 versus cluster 3 and 4 for most of the TF families. SOX-related factors and C/EBP-related family, on the other hand, seem to be distributed across several clusters. For SOX-related motifs, we observe that these motifs form 2 major subgroups as shown in Fig. 2c. SOX-related factors associated with PAM cluster 1 contain an extended binding sequence AACAATK**GCAKCAKTGTT** whereas those associated with PAM cluster 2 and 4 contain a shorter version AACAATG binding motif.

After this global analysis of the alteration patterns across all 512 motifs, we next focused on specific motifs related to transcription factors which play a role in cancer. We extracted from COSMIC cancer gene census the list of transcription factors to investigate how the binding motifs of transcription factors mutated in the cancer genome are affected by somatic mutations. We computed the differential motif alteration probabilities of 30 signatures for 40 TF motifs with the strongest differential impact (Additional file 2: Figure S2). This map displays two broad groups of transcription factors with opposite behavior. Some interesting patterns can be observed. For example, HNF1A, a liver-specific transcription factor, appears to have a strong excess of creation upon signature 12, which is specifically found in liver cancer. Given the high expression of HNF1A in liver tissue, this excess of new binding sites could result in some novel, functional binding sites in liver cancer.

In order to capture the complexity of the relation between the mutational signatures and the binding motifs, we applied SOM clustering to group motifs showing similar behaviors. We performed SOM clustering over the 192 possible mutational transitions (taking both creation and disruption into account). We also combined these mutational probabilities into the 30 mutational signatures.

**Fig. 3:**
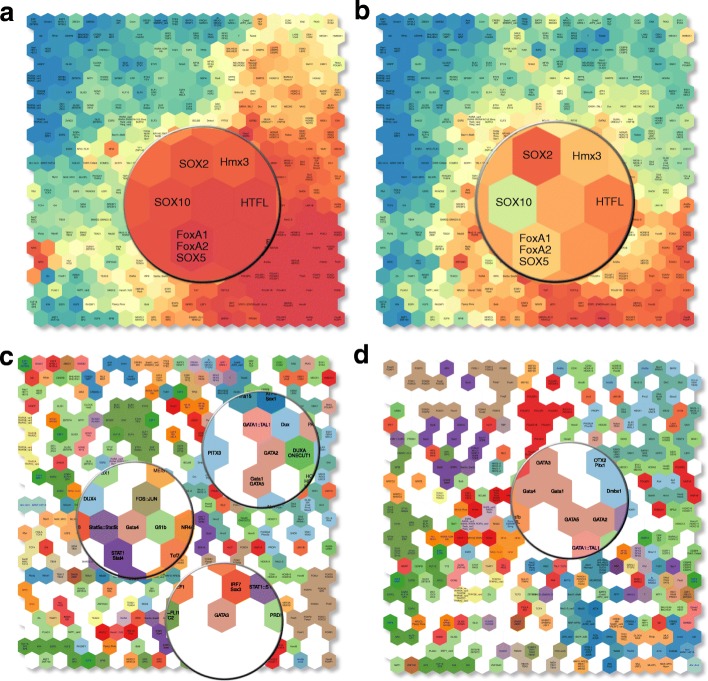
Transcription factor motifs of width 6 to 19: **a** SOM of Signature 10 creation probability; **b** SOM of Signature 23 motif creation probability; **c** SOM using 30 motif alteration signatures with TF family colour coded; **d** SOM using 96 motif alteration probabilities with TF family colour coded

The SOM clustering provides a clear picture on the similarity of the alteration behavior of transcription factors across all trinucleotide mutational probability and the 30 mutational signatures. Transcription factors of the same transcription factor families often share similar binding sequence and their motif alteration behavior should also be similar. We observe that overall, transcription factors of the same family are well clustered together and globally share similar motif alteration probability patterns (Fig. 3a,b). For example, the SOX transcription factor family with SOX2, SOX4, SOX8, SOX9, SOX11, SOX10, SOX11, SOX17, and SOX21 are clustered together. However, for specific signatures, differences between related transcription factors do exist. In Fig. 3a, SOX10 appears to have a very similar creation probability to its neighbor SOX2 for signature 10, but shows a much lower creation probability in signature 23 (Fig. 3b).

Overall, by coloring the cells according to the family of the TF, we observe a global clustering of motifs from the same structural class (Fig. 3c,d). However, this does not hold true for all families; we highlighted some members of the GATA-family in Fig. 3c which appear to be dispersed across the SOM map.

### Correlation Between Motif Creation and Disruption Signature

**Fig. 4:**
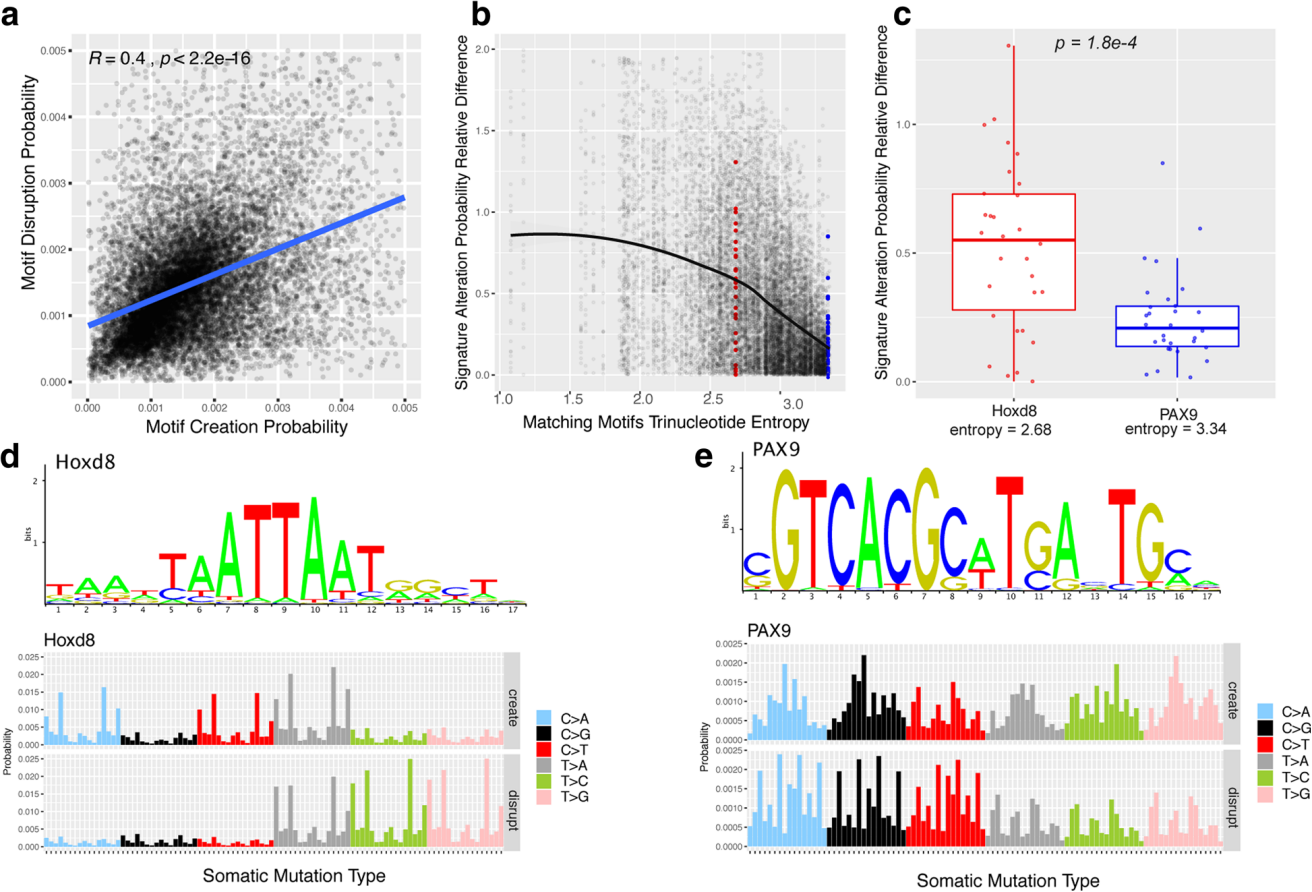
Demonstration of alteration correlation effect between creation and disruption probability: **a** Scatter plot of correlation between the creation and disruption probabilities for all 512 motifs across the 30 mutational signatures where each dot corresponds to a TF motif in one of the 30 signatures; **b** Difference of creation/disruption probabilities vs. motif entropy, with Hoxd8 highlighted in red and Pax9 in blue; **c** Boxplot of alteration probability absolute relative difference of Hoxd8 versus PAX9; **d** PWM logo of Hoxd8 (top) and alteration probability of Hoxd8 with respect to 96 trinucleotides (bottom); **e** PWM logo of PAX9 (top) and alteration probability of PAX9 with respect to 96 trinucleotides (bottom)

The previous results indicate that for many transcription factors, there is a compensating effect of creation and disruption. Further investigating this relationship we found indeed that the alteration probability between creation and disruption event in all signatures have globally a strong correlation between the creation and disruption (Fig. 4a). We found this correlation to be strongly related to the trinucleotide diversity of the motif matching sequences. To quantify this diversity, the trinucleotide content of each of the matching sequences is determined and the entropy is computed for each TF motif. The similarity between creation and disruption probability of each motif is evaluated using the absolute relative difference:

12 $$ {}\left| RD(s,tf)\right|=\frac{2\left|{Pr(s,tf,create) - Pr(s,tf,disrupt)}\right|}{ Pr(s,tf,create) + Pr(s,tf,disrupt)}  $$

where *s* represents a signature and *tf* a binding motif. The scatter plot in Fig. 4b shows an inverse relation between the complexity of the binding sequences (as measured by the entropy of these sequences) and the difference between creation and disruption. Hence, the more complex the motif content, the stronger the creation and the disruption signature probability are correlated.

This effect is illustrated using Hoxd8 and PAX9 as an example. To ensure that the motif width does not bias the results, both selected motifs have a width of 17 nucleotides. As illustrated in Fig. 4d and Fig. 4e, Hoxd8 and PAX9 have very different trinucleotide content. This is obvious looking at the motif logo. The motif alteration probability with respect to the 96 trinucleotide mutation is shown below the logos. For Hoxd8, the motif alteration probability concentrates on trinucleotide mutations related to TAA, AAT, TTA, and ATT where the overlap of these trinucleotide mutation creation and disruption only occurs on A[T >A]A, A[T >A]T, T[T >A]A, and T[T >A]T.

On the other hand, the alteration probability of PAX9 is distributed along all the 96 trinucleotide mutations, resulting in the creation and disruption probability to be strongly correlated along the 96 trinucleotide mutations. This translates into a high similarity across the 30 mutational signatures. This is shown in Fig. 4c for Hoxd8 and PAX9 across the 30 mutational signatures.

### Association of Deamination Signature and TFBS Creation

**Fig. 5:**
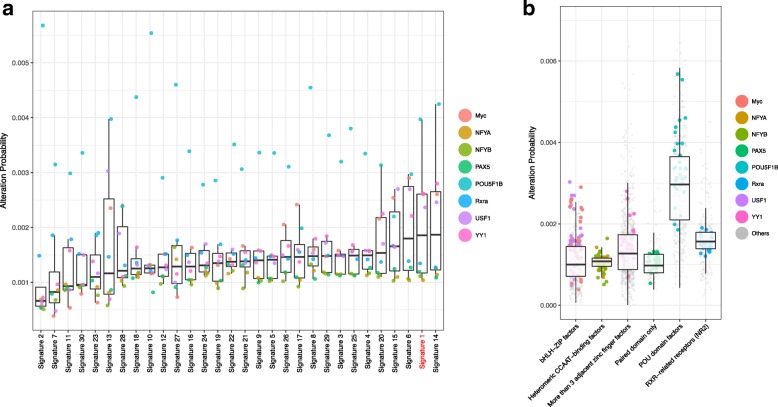
CpG deamination associated TF motif **a** creation probability for all 30 mutational signatures; **b** creation probability of all TF of the corresponding TF family

We next sought to validate our predictions using independent data. In [14], Zemojtel et al. described a set of transcription factors whose binding sites are frequently created as a result of CpG deamination process during evolution. These transcription factors include: c-Myc(Myc), Nfya, Nfyb, Oct4(POU5F1B), PAX5, Rxra, Usf1, and YY1. Given that some of the mutational signatures in cancer are related to CpG deamination, we sought to verify if the same transcription factors are impacted by this process due to somatic mutations. The creation probability of these transcription factor motifs are plotted in Fig. 5a over all 30 mutational signatures.

Signature 1 was described as being related to deamination of 5-methylcytosine [15] and ranks as the second highest signature in terms of creation frequency of these motifs in Fig. 5a. Signature 14 shows a very similar mutational profile with strong C →T bias. In addition, it is interesting to note that the POU5F1B motif has a high creation probability compared to other TFs across all signatures. This phenomenon is due to the high genomic frequency of motifs which differ from POU5F1B binding motifs by one mutation, which gives rise to a large number of k-mers which closely resemble the POU5F1B binding domain and serve as a substrate for TF motif creation events.

If we extend this single TFs to the family they belong to, we also observe a much higher creation probability for the other members of the POU-family, compared to the families of the other impacted TFs (Fig. 5b).

### Mutation associated Mechanisms

There are three main molecular mechanisms leading to single nucleotide mutations in cancer: i) defective DNA mismatch repair (MMR); ii) APOBEC activity; and iii) transcription-coupled nucleotide excision repair (NER). In the catalogue of mutational signatures, several signatures can be related to each of these processes. We wanted to investigate which transcription factor motifs are most impacted by these three different molecular mechanisms.

**Fig. 6:**
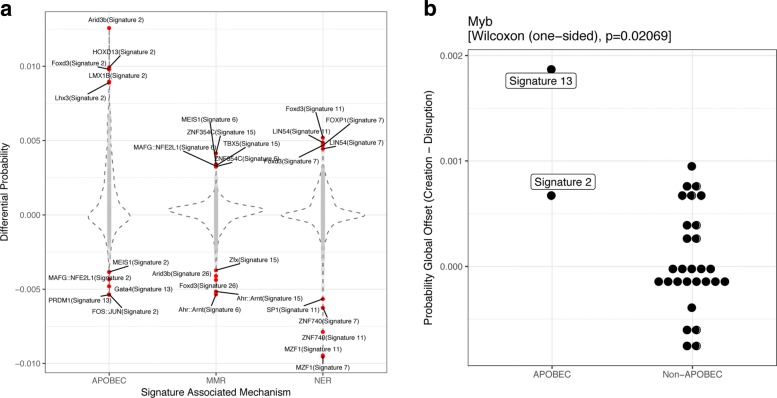
Multiple signatures driven molecular mechanisms in cancer with associated TF and the corresponding global alteration offset comparison: **a** Top ranked TF and signature pair for each of the 3 associated mechanism; **b** APOBEC associated signatures and Myb

For each of these mechanisms, we summarized the alteration results for all signatures annotated to the same mechanism; APOBEC is related to signatures 2 and 13, MMR to signature 6,15,20 and 26, and NER to signatures 4,7,11 and 22. We displayed the differential alteration probabilities (creation - disruption) in Fig. 6a. Interestingly, a number of transcription factor motifs show reverse behaviors with respect to these three mechanisms. For example, Foxd3 shows a much higher creation probability for APOBEC and NER related signatures, whereas the opposite holds for MMR signatures.

As an example, it was shown that a mutation associated with the APOBEC signature leads to the creation of a MYB binding site [16]. To understand if this single event leading to a driver mutation might result from a more general impact of the APOBEC signature on the binding sites of MYB, we indeed observed that the two APOBEC signatures (signature 2 and 13) show the highest bias towards MYB motif creation compared to all other signatures (P-value = 0.02, Fig. 6b). Hence, this single driver event might result from an elevated creation rate of potential MYB binding sites under these APOBEC signatures.

### Comparison with the PCAWG Dataset

We then sought to validate our predictions using a dataset of observed SNV in cancer genomes. We used a dataset of 2708 whole-genome sequencing covering 40 cancer subtypes. The idea of the validation is to compare the predicted frequencies of motifs alteration that are explained purely by the mutational signature and its biases towards certain trinucleotides, with the frequencies of motif alteration observed when considering a SNV dataset in cancer genomes. If the predicted and the observed frequencies are comparable, then most of the signal of motif creation or disruption can be explained by the impact of mutational signatures. If on the other hand we observe a difference, this discrepancy could be attributed to additional effects in the real dataset, such as positive or negative evolutionary pressure in the cancer dataset leading to an increased frequency of motif creation or disruption. Hence, our goal is to highlight such potential effects and to use our signature based prediction as a baseline.

**Fig. 7:**
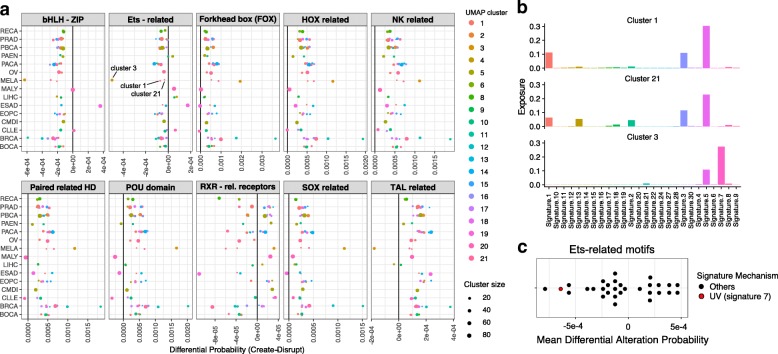
Inferred differential probability using exposure from the PCAWG dataset. **a** differential probability of creation minus disruption for all TF families with at least 10 members, across tumor subtypes containing at least 50 samples. The colored dots represent the samples of the different UMAP clusters, with size proportional to the number of these samples. **b** Mutational profile for the 3 signatures mostly represented within the melanoma (MELA) subtype. **c** Differential alteration probability (creation minus disruption) for all 30 signatures for the Ets-related family of motifs. UV-related signature 7 is highlighted in red

In order to compute a predicted alteration probability per patient, we used the signature exposure of each patient, and performed a linear combination based on the exposure values. In order to take into account the fact that there is heterogeneity between tumors even within a subtype, we used a clustering of samples based on their exposure to mutational signatures to split each subtype. While some subtypes are rather homogeneous i.e. lie mostly within one cluster, some others are spread across many clusters (see Additional file 3: Figure S3). The differential alteration probability is computed for each motif and each cluster within the subtypes by first subtracting the motif disruption and creation probability for each TF and then taking the median of the alteration difference by cancer entity and TF family. In Fig. 7a, we display the results for all TF families containing at least 10 motifs and each tumor subtype containing at least 50 samples.

For the 10 TF families, we observed that some appear to have a global excess of predicted disruption events over all cancer subtypes (bHLH, Ets-related), while other show the opositive effect (NK-related, SOX-related,...). Beyond these general trends, we have also observed that some cancer subtypes show a different trend. For example, Esophageal Adenocarcinoma (ESAD) shows an excess of bHLH-type motif creations, while melanoma (MELA) is predicted to contain an excess of Tal-related disruptions as opposed to all other cancer types. Looking even more in detail, differences within the tumor subtypes were obvious, between the samples belonging to the different clusters defined previously through the UMAP analysis. A prominent example was the alteration probabilities of Ets-related motifs in Melanoma. Melanoma shows by far the highest bias toward disruptions of Ets-motifs. However, this only holds true for the melanoma samples belonging to cluster 3 (brown in the figure). The other melanoma samples (cluster 1 and 21) show a balance between creation and disruption. These 3 melanoma clusters differ in their exposure profile (Fig. 7b). Indeed, cluster 3, which displays the high Ets disruption bias, is highly exposed to Signature 7, which is related to UV-induced mutations, and displays indeed the second highest disruption probability for Ets-motifs (Fig. 7c). The relation between UV-induced signatures and Ets binding sites has been described previously [17]. In this study, an increase of mutations in binding sites for TFs was described, in particular for Ets binding sites and UV induced mutations. Hence, it appears that our model is capable of capturing this effect, despite the fact that we focus on motifs occurrences and not actual binding sites.

**Fig. 8:**
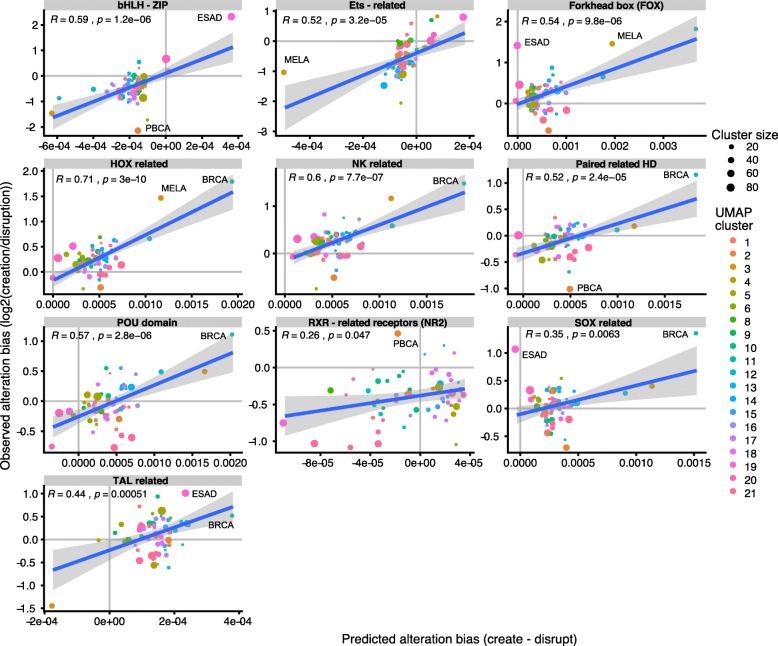
Comparison of the predicted differential alteration (creation minus disruption) (x-axis) with the observed log-ratio of motif creation counts versus the disruption counts, obtained from the SNV in each tumor subtype. Correlation coefficient and p-values for the regression are indicated in the maps. Color dots represent samples belonging to a tumor subtype and a UMAP cluster. Some interesting sample groups are indicated explicitely

Finally, we compared the predictions with the observed alterations across the PCAWG dataset. To perform this comparison, we first determined the observed motif creation and disruption probabilities of all TFs across all samples within a given cohort using the motif alteration calling pipeline described in the method section. This pipeline predicts, for each SNV, whether it disrupts or creates potential binding motifs, and yields for each patient and each motif a creation and disruption count for each TF motif. We computed the log-ratio of the number of observed creation/disruption events as determined by the pipeline, by summarizing all samples belonging to a tumor subtype and UMAP cluster. The comparison of this observed alteration bias with the alteration bias predicted by our model is displayed in Fig. 8. Overall, we found a very good correlation between the model prediction and the observation. The correlation between both was significant for all TF families, with some differences. For example, the correlation is very high for Ets-related factors, for which in most cases, the direction of the bias was concordant between model prediction and observed alteration counts. In other cases however, despite a good correlation, the direction of the alteration did not coincide well. For example, for RXR-related factors, we observe a global excess of motif disruptions, even for samples for which our model predicts an excess of creations. The prediction made for melanoma samples in cluster 3 of a strong excess of disruptions over creations is confirmed in the observed alterations.

## Discussion

The purpose of this work is to translate the patterns of mutational signatures observed in cancer genomes into patterns of alterations of motifs corresponding to transcription factor binding sites. For this purpose, we have established a theoretical framework to compute the probabilities of alterations (creation or disruption) over a large set of known motifs from the JASPAR database, by using a k-mer based approach. We observed distinct patterns of creation/disruption event, which are generally shared by the motifs belonging to the same TF family. However, despite this general agreement within a family, differences can be observed as can be seen for example for the GATA-family motifs highlighted in Fig. 3. This could possibly lead to a shift in the number of occurring binding sites from one transcription to a different one from the same family, and lead to a partial rewiring of the regulatory network.

This model only takes into account the effect of the mutational signatures and therefore determines an overall expected background of motifs alterations, in the absence of any further evolutionary mechanism like positive or negative selection. Departures from these expected patterns could be interpreted as the effect of additional specific mechanisms impacting the landscape of binding motifs. In this respect, the example of Ets motifs is interesting. We find an overall tendency towards an excess of disruption of motifs over creation of novel motifs across many tumor types, especially in melanoma. This is also confirmed by the overall observed patterns of motif breaks due to single-nucleotide somatic variants. In melanoma, this fits the described excess of mutations within Ets binding sites due to UV radiation [17]. In accordance with this, we only observe this strong disruption bias for tumor samples within a specific cluster strongly impacted by the UV-light signature, but not in the other melanoma samples. This seems to indicate that, despite being restricted to actual Ets-binding sites, which are way less abundant that Ets motifs, the overall signature seems to capture this effect. However, the most prominent non-coding regulatory mutation described so far, affecting Ets-binding is actually a creation of novel binding sites within the *TERT* promoter region [1, 2]. Hence, we have an overall genome-wide excess of Ets-motif disruption, but a focal appearance of novel Ets-binding sites. We have previously shown that this tendency towards creation of novel Ets binding motifs is found in other gene promoters, like *BCL2* in lymphoma or *NEAT1* in liver cancer [4]. These are also driving changes in the expression of the corresponding gene (especially *TERT* and *BCL2*), highlighting the potential functional significance of these focal events.

Very few non-coding driver mutations have been found in the extensive PCAWG study [18]. Beyond the *TERT* promoter mutation, a number of recurring promoter mutations have been found, however it is unclear whether they might have a functional impact given the lack of association with gene expression change (e.g. *PAX5*). We also expect that the vast majority of the alteration patterns that we describe here will have a low if any functional impact individually. These would be classically defined as passenger mutations, which are usually discarded in cancer studies. However, our recent study has highlighted that the mutational load of so-called passenger mutations might contribute globally to a functional impact and contribute to the cancer phenotype [4]. Hence, describing the global pattern of motif alterations induced by mutational signatures sheds a new light onto the potential impact of mutational signature in shaping the global mutational load of somatic mutations in cancer genomes.

## Conclusion

In this study, we investigated the theoretical impact of cancer mutational signatures on regulatory elements of the non-coding genome and provided an outline of a Bayesian framework for motif alteration analysis using mutational signatures. One of the key finding in this study is the correlation effect between motif creation and motif disruption. The correlation between the motif alteration probability was found to be strongly positively associated to the motif entropy. Further, previously described effects such as the impact of specific signatures on families of transcription factors can be reproduced by our theoretical model. An intriguing finding was that the described non-coding driver leading to MYB creation in T-ALL could be related to a global increased creation probability in APOBEC driven cancer types. Finally, the motif alteration signatures were used to infer the alteration events of each PCAWG cohort using the corresponding signature exposure. We confirmed that Ets-motifs in melanoma display a strong excess of motif disruptions over novel motif creations, especially for the samples exposed to the UV-induced signature. This is predicted by our model and validated from the actual SNV disruption counts. Therefore, this motif alteration signature can serve as a background model for point mutation analysis for large datasets.

## Additional file

Additional file 1 Motif alteration signatures heatmap where the vertical axis corresponds to the 30 mutational signatures and the horizontal axis corresponds to the 512 motifs in JASPAR database. A *k*_*clust*_=4 PAM clustering performed over the TF alteration probability. The TF family is indicated as a colored bar on the right-hand side. (pdf 199 kb)

Additional file 2 Differential motif alteration (creation minus disruption) heatmap of TF in COSMIC cancer gene census. Shown are the 40 TFs with the highest differential probability accross at least 3 signatures. (pdf 28 kb)

Additional file 3 (top) UMAP representation of the 2708 WGS samples from PCAWG, according to their exposure to the mutational signatures. Colors indicate the tumor subtype. (bottom) Clustering of the UMAP map using hdbscan. The number of samples within each cluster and their tumor subtype is indicated as a barplot (bottom right). (pdf 245 kb)

Additional file 4 Pseudo-code to compute the conditional alteration probability. (pdf 104 kb)

Additional file 5 List of abbreviations used in the PCAWG data for tumor subtypes. (pdf 36 kb)

Additional file 6 Excel sheet containing the predicted alteration frequencies for all JASPAR motifs for all 30 COSMIC mutational signatures. (xlsx 1977 kb)

## Abbreviations

ICGC: International cancer genome consortium
MMR: DNA mismatch repair
NER: Nucleotide excision repair
PAM: Partition around medoids
PCAWG: Pan-cancer analysis of whole genomes
PWM: Position weight matrix
RSAT: Regulatory sequence analysis toolbox
SNV: Single-nucleotide variants
SOM: Self-organizing maps
TF: Transcription factor
TFBS: Transcription factor binding site
UMAP: Uniform manifold approximation and projection
UV: Ultraviolet
WES: Whole exome sequencing
WGS: Whole genome sequencing
