# Supplementary figures and images for: Impact of cancer mutational signatures on transcription factor motifs in the human genome
Source: BMC Med Genomics. 2019 May 20;12:64. doi: 10.1186/s12920-019-0525-4 (PMC6528224; doi:10.1186/s12920-019-0525-4)

Creation

Disruption

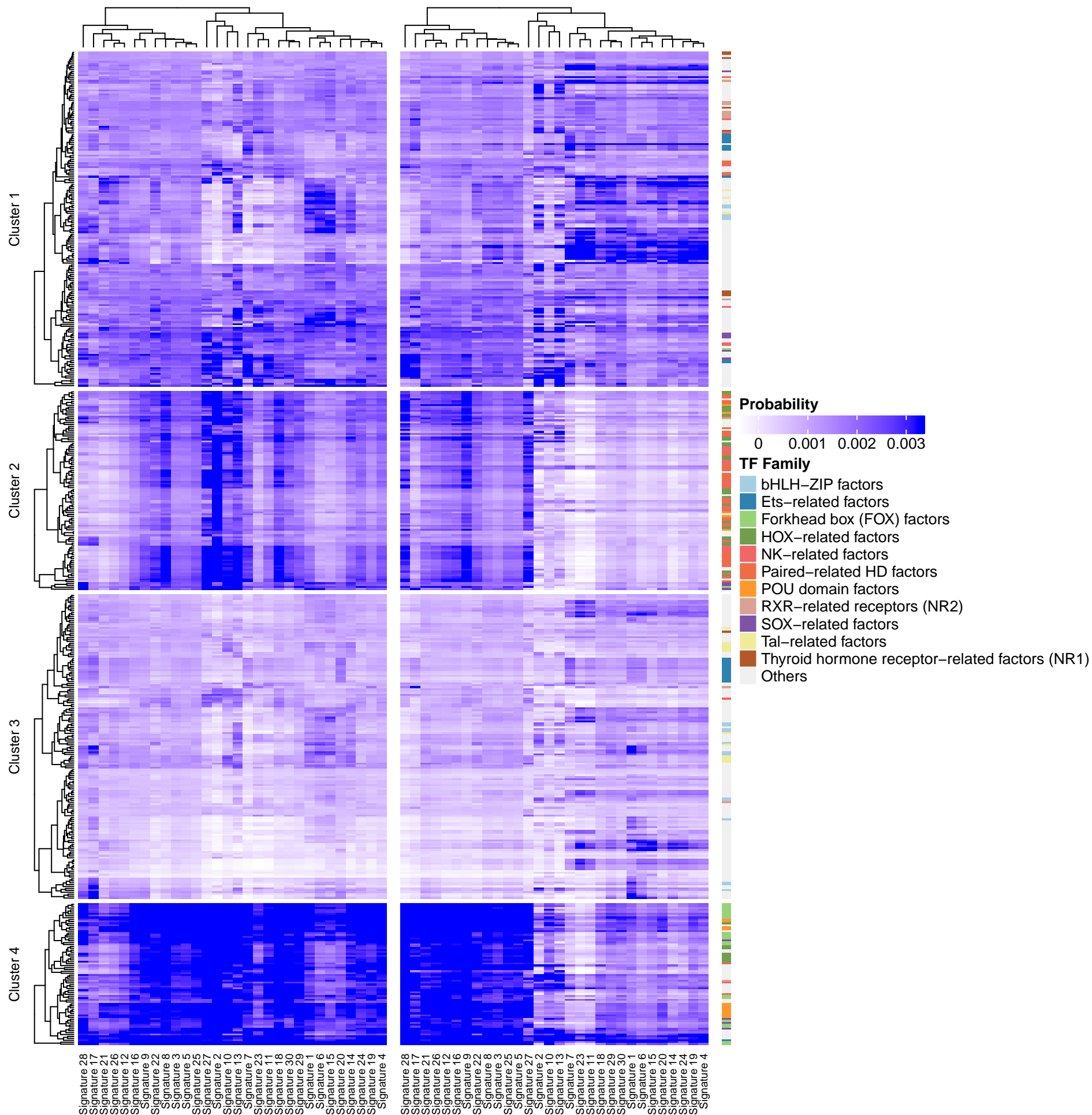

Supplement: Supplementary file 1 — Motif alteration signatures heatmap where the vertical axis corresponds to the 30 mutational signatures and the horizontal axis corresponds to the 512 motifs in JASPAR database. A kclust=4 PAM clustering performed over the TF alteration probability. The TF family is indicated as a colored bar on the right-hand side. (pdf 199 kb) [file 12920_2019_525_MOESM1_ESM.pdf]

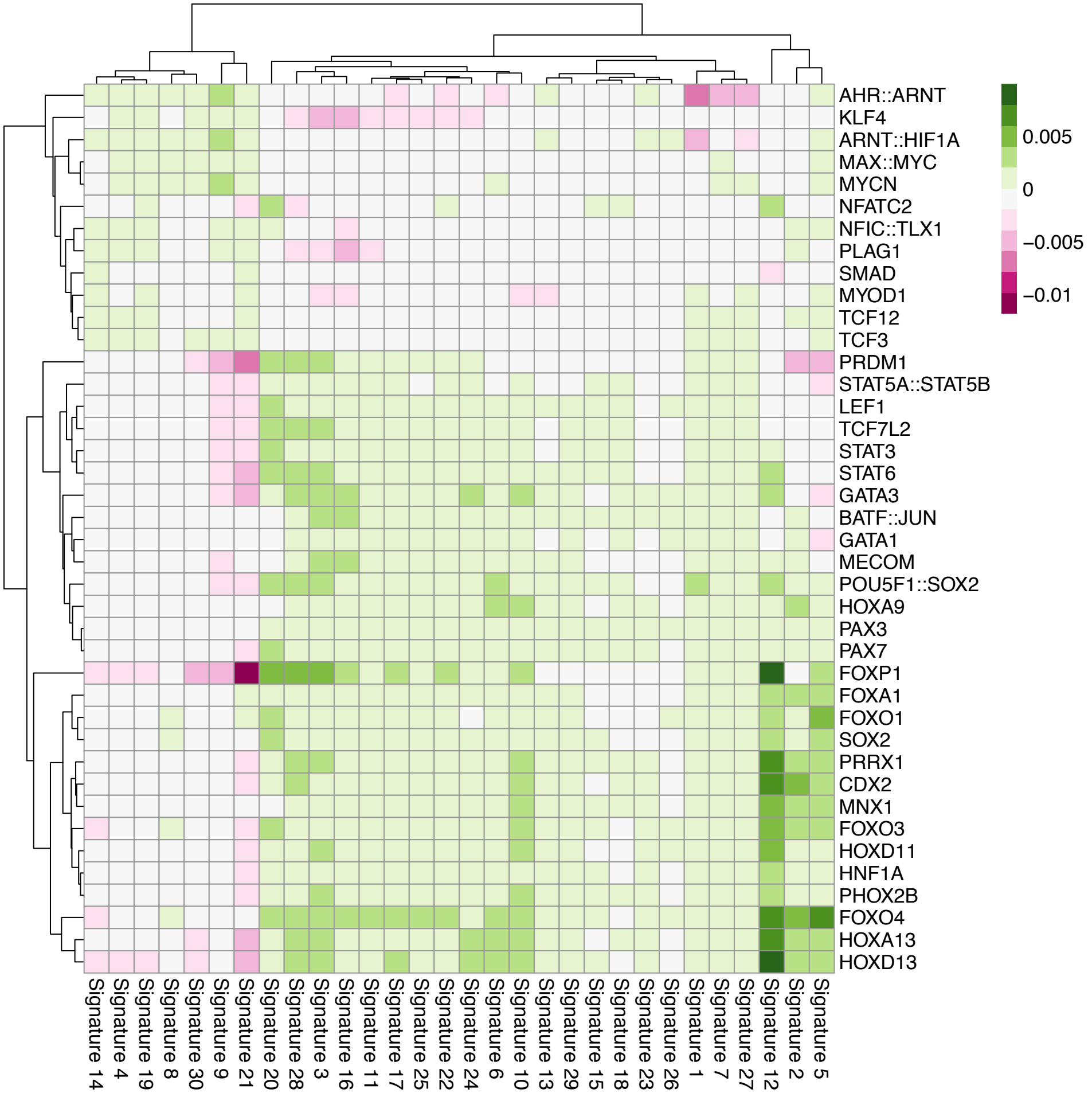

Supplement: Supplementary file 2 — Differential motif alteration (creation minus disruption) heatmap of TF in COSMIC cancer gene census. Shown are the 40 TFs with the highest differential probability accross at least 3 signatures. (pdf 28 kb) [file 12920_2019_525_MOESM2_ESM.pdf]
