# Supplementary material for: Impact of cancer mutational signatures on transcription factor motifs in the human genome
Source: BMC Med Genomics. 2019 May 20;12:64. doi: 10.1186/s12920-019-0525-4 (PMC6528224; doi:10.1186/s12920-019-0525-4)
Supplement: Supplementary file 3 — (top) UMAP representation of the 2708 WGS samples from PCAWG, according to their exposure to the mutational signatures. Colors indicate the tumor subtype. (bottom) Clustering of the UMAP map using hdbscan. The number of samples within each cluster and their tumor subtype is indicated as a barplot (bottom right). (pdf 245 kb) [file 12920_2019_525_MOESM3_ESM.pdf]

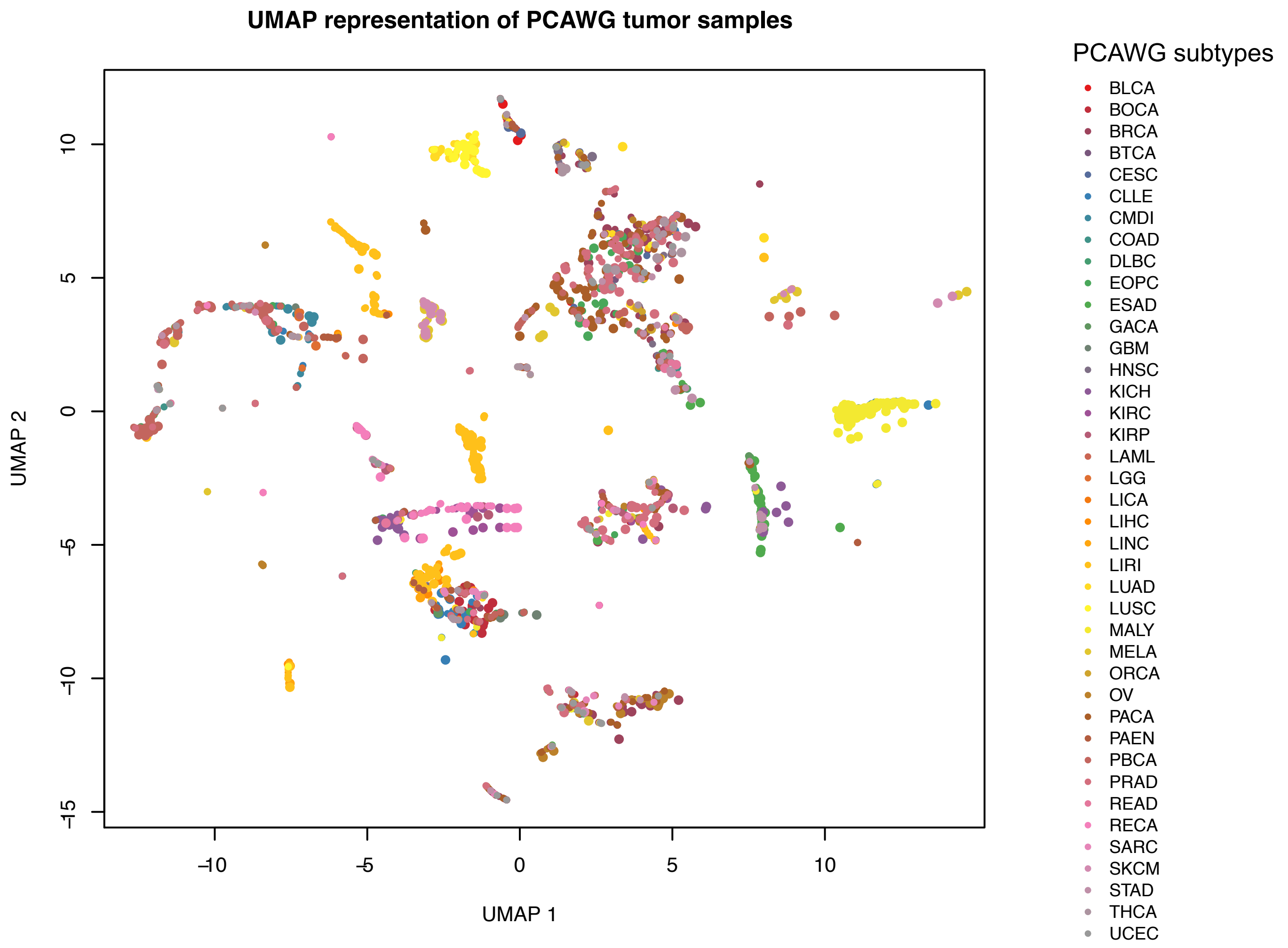
UMAP representation of PCAWG tumor samples
UMAP 1
UMAP 2
PCAWG subtypes
BLCA
BOCA
BRCA
BTCA
CESC
CLLE
CMDI
COAD
DLBC
EOPC
ESAD
GACA
GBM
HNSC
KICH
KIRC
KIRP
LAML
LGG
LICA
LIHC
LINC
LIRI
LUAD
LUSC
MALY
MELA
ORCA
OV
PACA
PAEN
PBCA
PRAD
READ
RECA
SARC
SKCM
STAD
THCA
UCEC

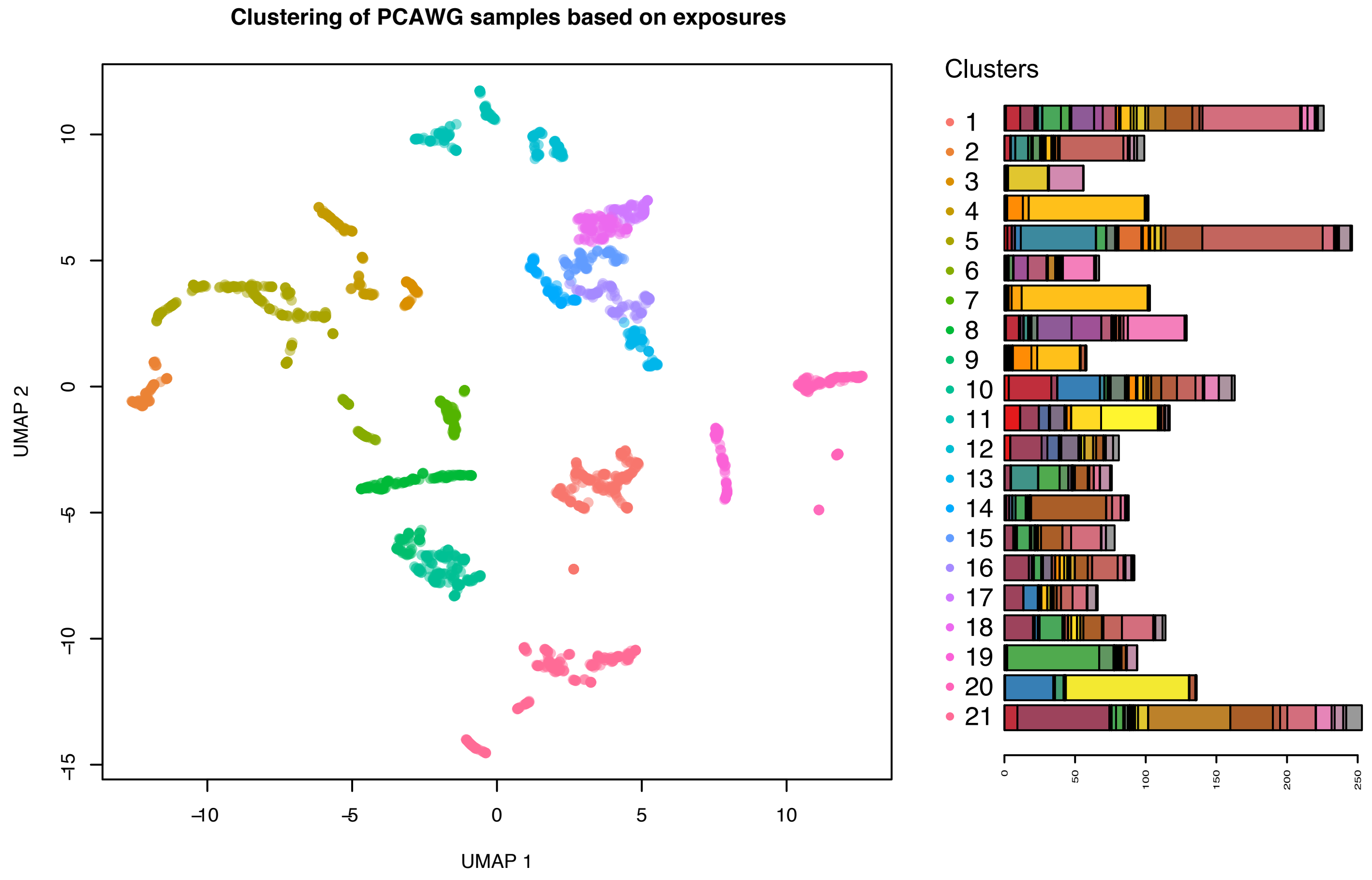
Clustering of PCAWG samples based on exposures
UMAP 1
UMAP 2
Clusters
1
2
3
4
5
6
7
8
9
10
11
12
13
14
15
16
17
18
19
20
21
