# Supplementary material for: Impact of cancer mutational signatures on transcription factor motifs in the human genome
Source: BMC Med Genomics. 2019 May 20;12:64. doi: 10.1186/s12920-019-0525-4 (PMC6528224; doi:10.1186/s12920-019-0525-4)
Supplement: Supplementary file 4 — Pseudo-code to compute the conditional alteration probability. (pdf 104 kb) [file 12920_2019_525_MOESM4_ESM.pdf]

TF PWM Alteration Probability Estimation Algorithm

To summarize, the alteration probability estimation algorithm consist of the following steps:

1. **Scan all k-mer sequences**

   For $i = 1$ to I, where i is the index of transcription factor set PWM

   Obtain k, where k is the width of pwm(i)

   For $p_k = 1$ to P_k, where p_k is the index of k-mer sequence set SEQ

   Match pwm(i) against seq(p_k)

   If p-value > 0.001

   add seq(p_k) to MatchSEQ set

2. **Counting every alteration event**

   For $i = 1$ to I, where i is the index of transcription factor set PWM

   For $j = 1$ to J, where j is the index of element in MatchSEQ

   For $q_{trinuc} = 1$ to Q_{trinuc}, where q_{trinuc} is the index of the 96 mutation type

   For $n_{pos} = 1$ to $(k-2)$, where n_{pos} is the position in sequence matchseq$_j$

   If matchseq$_j[n_{pos} : n_{pos} + 2] == mut(q_{trinuc}, ref)$

   mutseq = matchseq$_j$

   mutseq$[n_{pos} : n_{pos} + 2] = mut(q_{trinuc}, alt)$

   If mutseq not in matchseq$_j$

   $count_{mut}(pwm(i), mut(q_{trinuc}), disrupt) += count_{seq}(matchseq_j)$

   $count_{mut}(pwm(i), mut(q_{trinuc}), create) += count_{seq}(mutseq)$

3. **Normalize probability**

   For $i = 1$ to I, where i is the index of transcription factor set PWM

   For $q_{trinuc} = 1$ to Q_{trinuc}, where Q_{trinuc} is the index of the 96 mutation types

$$p_{mut}(pwm(i), mut(q_{trinuc}), disrupt) = \frac{count_{mut}(pwm(i), mut(q_{trinuc}), disrupt)}{count_{genome}(k, mut(q_{trinuc}, ref))}$$

$$p_{mut}(pwm(i), mut(q_{trinuc}), create) = \frac{count_{mut}(pwm(i), mut(q_{trinuc}), create)}{count_{genome}(k, mut(q_{trinuc}, ref))}$$

Bayesian Inference of Transcription Factor Signature Alteration Probability

To compute the transcription factor signature alteration probability $Pr(a|s_i, tf_k)$, we have:

$$Pr(a|s_i, tf_k) = \sum_{j=1}^{96} Pr(a, m_j|s_i, tf_k) \tag{1}$$

Based on the Bayesian tree described in Fig 1c, we have the joint probability of all parameters described by Eq. 2.

$$\begin{aligned}
Pr(a, tf_k, m_j, s_i) &= Pr(a, tf_k|m_j)Pr(m_j|s_i)Pr(s_i) \qquad (2)\\
Pr(a, tf_k, m_j|s_i) &= Pr(a, tf_k|m_j)Pr(m_j|s_i)\\
\frac{Pr(a, tf_k, m_j|s_i)}{Pr(tf_k)} &= \frac{Pr(a, tf_k|m_j)}{Pr(tf_k)} \cdot Pr(m_j|s_i)\\
Pr(a, m_j|s_i, tf_k) &= Pr(a|m_j, tf_k)Pr(m_j|s_i) \qquad (3)
\end{aligned}$$

Combining Eq. 3 and Eq. 1, we have Eq.(5) in the main text.
