# Supplementary material for: Impact of cancer mutational signatures on transcription factor motifs in the human genome
Source: BMC Med Genomics. 2019 May 20;12:64. doi: 10.1186/s12920-019-0525-4 (PMC6528224; doi:10.1186/s12920-019-0525-4)
Supplement: Supplementary file 5 — List of abbreviations used in the PCAWG data for tumor subtypes. (pdf 36 kb) [file 12920_2019_525_MOESM5_ESM.pdf]

List of abbreviations for cancer types

ACC	Adrenocortical carcinoma
BLCA	Bladder Urothelial Carcinoma
BOCA	Bone Cancer
BRCA	Breast invasive carcinoma
CESC	Cervical squamous cell carcinoma and endocervical adenocarcinoma
CHOL	Cholangiocarcinoma
CLLE	Chronic Lymphocytic Leukemia
CMDI	Chronic Myeloid Disorders
COAD	Colon adenocarcinoma
COADREAD	Colorectal cancer
DLBC	Lymphoid Neoplasm Diffuse Large B-cell Lymphoma
EOPC	Early Onset Prostate Cancer
ESAD	Esophageal Adenocarcinoma
ESCA	Esophageal carcinoma
GBM	Glioblastoma multiforme
HNSC	Head and Neck squamous cell carcinoma
KICH	Kidney Chromophobe
KIRC	Kidney renal clear cell carcinoma
KIRP	Kidney renal papillary cell carcinoma
LAML	Acute Myeloid Leukemia
LGG	Brain Lower Grade Glioma
LICA	Liver Cancer
LIHC	Liver hepatocellular carcinoma
LINC	Liver Cancer
LIRI	Liver Cancer
LUAD	Lung adenocarcinoma
LUSC	Lung squamous cell carcinoma
MALY	Malignant Lymphoma
MESO	Mesothelioma
NBL	Neuroblastoma
ORCA	Oral Cancer
OV	Ovarian serous cystadenocarcinoma
PAAD	Pancreatic adenocarcinoma
PACA	Pancreatic Cancer
PAEN	Pancreatic Cancer Endocrine neoplasms
PBCA	Pediatric Brain Cancer
PCPG	Pheochromocytoma and Paraganglioma
PRAD	Prostate adenocarcinoma
READ	Rectum adenocarcinoma
RECA	Renal Cancer
SARC	Sarcoma
SKCM	Skin Cutaneous Melanoma
STAD	Stomach adenocarcinoma
TGCT	Testicular Germ Cell Tumors
THCA	Thyroid carcinoma

THYM	Thymoma
UCEC	Uterine Corpus Endometrial Carcinoma
UCS	Uterine Carcinosarcoma
UVM	Uveal Melanoma
